# Body growth assessment in children with IgE-mediated cow's milk protein allergy fed with a new amino acid-based formula

**DOI:** 10.3389/falgy.2022.977589

**Published:** 2022-09-05

**Authors:** Rita Nocerino, Serena Coppola, Laura Carucci, Lorella Paparo, Anna Fiorenza De Giovanni Di Santa Severina, Roberto Berni Canani

**Affiliations:** ^1^ Department of Translational Medical Science, University of Naples Federico II, Naples, Italy; ^2^ ImmunoNutritionLab at CEINGE Advanced Biotechnologies, University of Naples Federico II, Naples, Italy; ^3^ European Laboratory for the Investigation of Food-Induced Diseases, University of Naples Federico II, Naples, Italy; ^4^ Task Force on Microbiome Studies, University of Naples Federico II, Naples, Italy

**Keywords:** anthropometric measurements, hypoallergenic formula, food allergy, diet therapy

## Abstract

**Background:**

Amino acid-based formula (AAF) is a relevant dietary option for non-breastfed children. The present study was designed to evaluate the body growth pattern in cow's milk protein allergy (CMPA) children treated for 6 months with a new AAF.

**Methods:**

This was an open-label, single arm study evaluating body growth pattern in immunoglobulin E (IgE)-mediated CMPA infants receiving a new AAF for 6 months. The outcomes were anthropometry (weight, length, head circumference), adherence to the study formula and occurrence of adverse events (AEs).

**Results:**

Fifteen children [all Caucasian and born at term; 53.3% born with spontaneous delivery; 80% male; 80% with familial allergy risk; mean age (±SD) 3 ± 2.5 months at IgE-mediated CMPA diagnosis; mean age (±SD) 16.7 ± 5.9 months at enrolment, mean total serum IgE (±SD) 298.2 ± 200.4 kU/L] were included and completed the 6-month study. Data from fifteen age- and sex-matched healthy controls were also adopted as comparison. At baseline, all CMPA patients were weaned and were receiving the new AAF. All 15 patients completed the 6-month study period. For the entire CMPA pediatric patients’ cohort, from baseline to the end of the study period, the body growth pattern resulted within the normal range of World Health Organization (WHO) growth references and resulted similar to healthy controls anthropometric values. The formula was well tolerated. The adherence was optimal and no AEs related to AAF use were reported.

**Conclusions:**

The new AAF ensured normal growth in subjects affected by IgE-mediated CMPA. This formula constitutes another suitable safe option for the management of pediatric patients affected by CMPA.

## Introduction

Cow's milk protein allergy (CMPA) is a relevant problem worldwide with lifelong implications for health. With an estimated prevalence up to 3% it is one of the most common food allergies and one of the main causes of food-induced anaphylaxis in the pediatric age (1). The mainstay of the CMPA treatment is the elimination from the diet of cow's milk proteins. If breastfeeding is not available, the child must be fed with a special formula adapted to CMPA dietary management. This formula must be adequate in terms of allergic and nutritional safety. The most used are the following: extensively hydrolysed whey or casein formulas, soy formulas, hydrolysed rice formulas or amino acid- based formulas (AAF) (2). Only the AAF provide nitrogen equivalent proteins as free amino acids, which cannot lead to any immune stimulation. Therefore, they are the only special formulas that are considered completely non-allergenic and are commonly considered as the safest dietary strategy for CMPA children (3)*.* For this reason, AAF are considered the first dietary choice for patients with anaphylaxis or with severe forms of CMPA at onset, or with multiple food allergies with growth faltering (3, 4–11). We have recently demonstrated the hypoallergenicity of a new AAF in children with immunoglobulin-E (IgE)-mediated CMPA (12). As more evidence is emerging that children with CMPA could have an increased risk of developing impaired body growth (13–19), as a part of our research project we also investigated the effects of the new AAF on body growth in pediatric IgE-mediated CMPA patients.

## Methods

**Figure 1:**
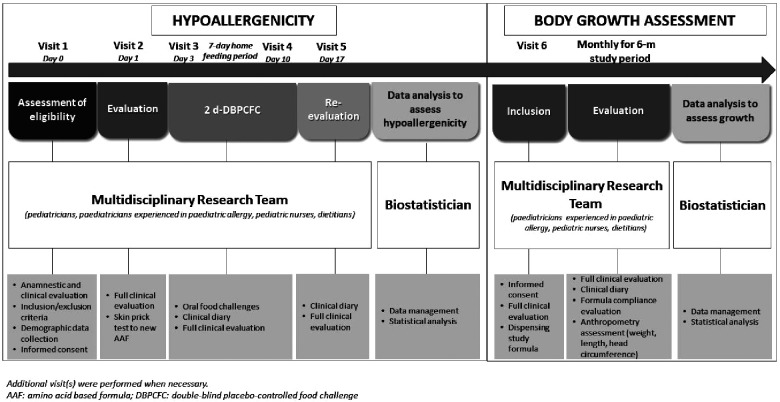
The design of the study.

The research project was conducted from March 2019 to March 2020, and as depicted in Figure 1 consisted in two subsequent phases.

### Study population

We considered for the research project, all pediatric patients, aged 1–36 months, consecutively observed at our tertiary centre for pediatric allergy with a sure diagnosis of IgE-mediated CMPA confirmed by the results of a double-blind, placebo-controlled food challenge (DBPCFC) performed in the previous 12 weeks*.* We excluded subjects aged <1 month and >36 months, breastfed infants, evidence of non IgE-mediated CMPA, chronic systemic diseases, congenital cardiac defects, acute or chronic infectious diseases, autoimmune diseases, immunodeficiencies, chronic inflammatory bowel diseases, celiac disease, cystic fibrosis, genetic and metabolic diseases, malignancies, chronic pulmonary diseases, malformations of the gastrointestinal and/or respiratory and/or urinary tract, use of systemic antibiotics or anti-mycotic drugs during 4 weeks before study entry, presence of CMPA-related symptoms in the previous 2 weeks, investigator's uncertainty about the willingness or ability of the subject to comply with the protocol requirements, and participation in any other studies involving investigational or marketed products concomitantly or within two weeks prior to entry into the study.

At baseline, the written informed consent was obtained from the parents/tutors of each study subject. At enrolment, anamnestic, demographic, anthropometric, and clinical data (including data related to CMPA), as well as information on sociodemographic factors, were obtained from the parents of each child, and collected in a specific clinical chart. All these variables were assessed by a multidisciplinary team composed by pediatricians, pediatric allergists, pediatric nurses, and dietitians.

As previously described (12), the skin prick test (SPT) with the new AAF was performed and subsequently, the patients underwent the DBPCFC with the new AAF or the placebo formula (namely, the formula previously given to the child as part of the child's successful elimination diet before study inclusion) introduced in a random order. The composition of the new AAF is described in Table 1. The new AAF (Sineall, Humana Italia S.*p*.A) complies with the American and European regulation in force at the start of the study (20, 21). Consecutive 29 pediatric patients [all Caucasian, 55.2% male, mean age (±SD) 16.9 ± 5.7 months] resulted negative to the SPT and DBPCFC with the new AAF, supporting the hypoallergenicity of the new AAF (12).

**Table 1:** Composition of the study formula.

		100 g	100 ml at 13% w/v
Calories	**kJ**	**1974**	**256**
	**kcal**	**471**	**61**
Total fat	**g**	**21** **.** **0**	**2** **.** **7**
Saturated fat	g	7.8	1.0
Monounsaturated fat	g	9.8	1.3
Polyunsaturated fat	g	3.0	0.4
Total carbohydrate	**g**	**58** **.** **2**	**7** **.** **6**
Sugars	g	0.0	0.0
Protein	**g**	**12** **.** **2**	**1** **.** **6**
Salt	**g**	**0** **.** **41**	**0** **.** **05**
Minerals			
Sodium	mg	165	21
Potassium	mg	570	74.1
Chloride	mg	300	39
Calcium	mg	460	59.8
Phosphorus	mg	295	38.4
Magnesium	mg	42	5.46
Iron	mg	6.9	0.90
Zinc	mg	7.1	0.92
Copper	g	420	55
Iodine	g	99	12.9
Manganese	mg	0.41	0.05
Fluorine	mg	0.3	0.04
Molybdenum	g	14.5	1.9
Chromium	g	14.5	1.9
Selenium	g	9.0	1.2
Vitamins			
Vitamin A	µg RE	550	71.5
Vitamin D	µg	8.0	1.0
Thiamin	mg	0.5	0.065
Riboflavin	mg	0.8	0.10
Niacin	mg	5.4	0.70
Vitamin B6	mg	0.7	0.09
Pantothenic Acid	mg	3.0	0.39
Biotin	µg	20	2.6
Folic Acid	µg	75	9.75
Vitamin B12	µg	2.1	0.27
Vitamin C	mg	63	8.2
Vitamin K	µg	60	7.8
Vitamin E	mg	10	1.3
Other nutrition facts			
Choline	mg	98	13
Inositol	mg	20	2.6
L-carnitine	mg	17.9	2.3
Taurin	mg	40	5.2
Linoleic Acid (LA)	mg	2900	377
α-linolenic Acid (ALA)	mg	294	38
Maltodextrins	g	42.3	5.5
Nucleotides			
Adenosine-5′-monophosphate	mg	6.9	0.9
Cytidine-5′-monophosphate	mg	3.8	0.5
Guanosine-5′-monophosphate	mg	1.3	0.2
Inosine-5′-monophosphate	mg	2.5	0.3
Uridine-5′-monophosphate	mg	4.5	0.6
Osmolarity	mOsmol/L	216	

The composition of the new amino acid-based formula was fully in line with the composition of other commercially available amino acid based formulas and with the actual recommendation for energy requirement provided by European Food Safety Authority (reference #37).

For the second phase of the research project, that we describe here, we randomly selected 15 CMPA patients from this cohort to evaluate the effects of this new AAF on body growth in a prospective 6-month study. Parents were invited to continue the use of the new AAF.

The outcomes were anthropometry (weight, length, head circumference), adherence to the study formula, and occurrence of adverse events (AEs).

### Procedures

All study subjects were evaluated monthly during the 6-month study period. During the visits anthropometric and clinical data, as well as possible occurrence of AEs were assessed by the previously described multidisciplinary team. At the baseline, and then monthly, all study subjects underwent a personalized dietary counselling session on how to follow an adequate cow milk protein-free diet (22). At each visit, study formula was dispensed to the parents of the CMPA patients. The recommended average of AAF consumption ranged between 200 and 500 ml/day (15–50% of total energy intake), according to patient's age and sex.

Parents were asked to record in a diary the daily formula and solid foods intake. Parents received complete oral and written instructions about how to weigh formula and solid foods, and how to record data in the diary. Formula adherence was evaluated by counting and weighing the returned tins and by reviewing the notes on the diary recorded by parents. Adherence was judged optimal in the presence of >80% recommended formula intake.

Anthropometric measurements were collected following standardized procedures. Briefly, naked subjects were weighed twice on calibrated electronic scales (Seca 834) or on mechanical scale (Seca 711) for later ages for all time points thereafter. Supine length of infants was measured twice using a standard measuring board (Seca 210 Mobile Measuring mat), while standing height measurements using standardised stadiometers were allowed from 24 months onwards. To measure head circumference (HC, the largest occipitofrontal circumference), a non-stretchable measuring tape was used in duplicate. If the anthropometric measures deviated substantially (>100 g for weight and >5 mm for length and head circumference), a third measurement was obtained.

As comparison, we used anthropometric data from a database of healthy children followed at the center for vaccination program available at the Center. From this database we randomly selected data from 15 age- and sex-matched healthy controls. Weight-for-age z-score (WAZ), length-for-age z-score (LAZ) and HC-for-age z-score (HCAZ) were calculated based upon the World Health Organization (WHO) Child Growth Standards (23) using the WHO Anthro Software *(available at* http://www.who.int/childgrowth/software/en/*)*.

Adverse events, serious and non-serious, during the 6-month study period were notified by the investigators and coded by diagnosis, severity, date of onset, and resolution. They were reported and classified as related (definitely, probably, or possibly related) or unrelated (unlikely or not related) based on a relationship to study formula intake according to the investigators.

All data were collected in the specific clinical chart.

### Data management and statistical analysis

All data were recorded anonymously. At the study Center, designated investigators were required to enter all collected data in the case report form (CRF). Two researchers performed separate checks of data completeness, clarity, consistency, and accuracy, and instructed site personnel to make any required corrections or additions. Using a single data-entry method, all data recorded in the CRF were entered in the study database by the same researcher. Then, the study dataset was reviewed and underwent data cleaning and verification according to standard procedures. Finally, the database was locked once it was declared complete and accurate, and the statistical analysis was performed by a statistician using SPSS for Windows (SPSS Inc, version 23.0, Chicago, IL).

The Kolmogorov-Smirnov test was used to determine whether variables were normally distributed. Descriptive statistics were reported as the means and standard deviations for continuous variables, and discrete variables were reported as the number and proportion of subjects with the characteristic of interest. Due to the varying ages of the subjects recruited to the study, growth parameters were converted to z-scores to allow for a meaningful comparison of the ability of the study formula to promote growth. Growth measures were presented as mean of z-score ± SD, and data between follow-up visits and baseline were compared using paired Student's *t*-test.

The level of significance for all statistical tests was two-sided, *p* < 0.05.

### Ethics

The study protocol, the subject information sheet, the informed consent form, and the clinical chart were reviewed and approved by the Ethics Committee of the University of Naples Federico II. The study was conducted in accordance with the Helsinki Declaration (Fortaleza revision 2013), the Good Clinical Practice Standards (CPMP/ICH/135/95), and the current Decree-Law 196/2003 regarding personal data and all the requirements set out in the European regulations on this subject. The study was registered in the ClinicalTrials.gov Protocol Registration System with the ID number NCT03909113.

## Results

**Figure 2:**
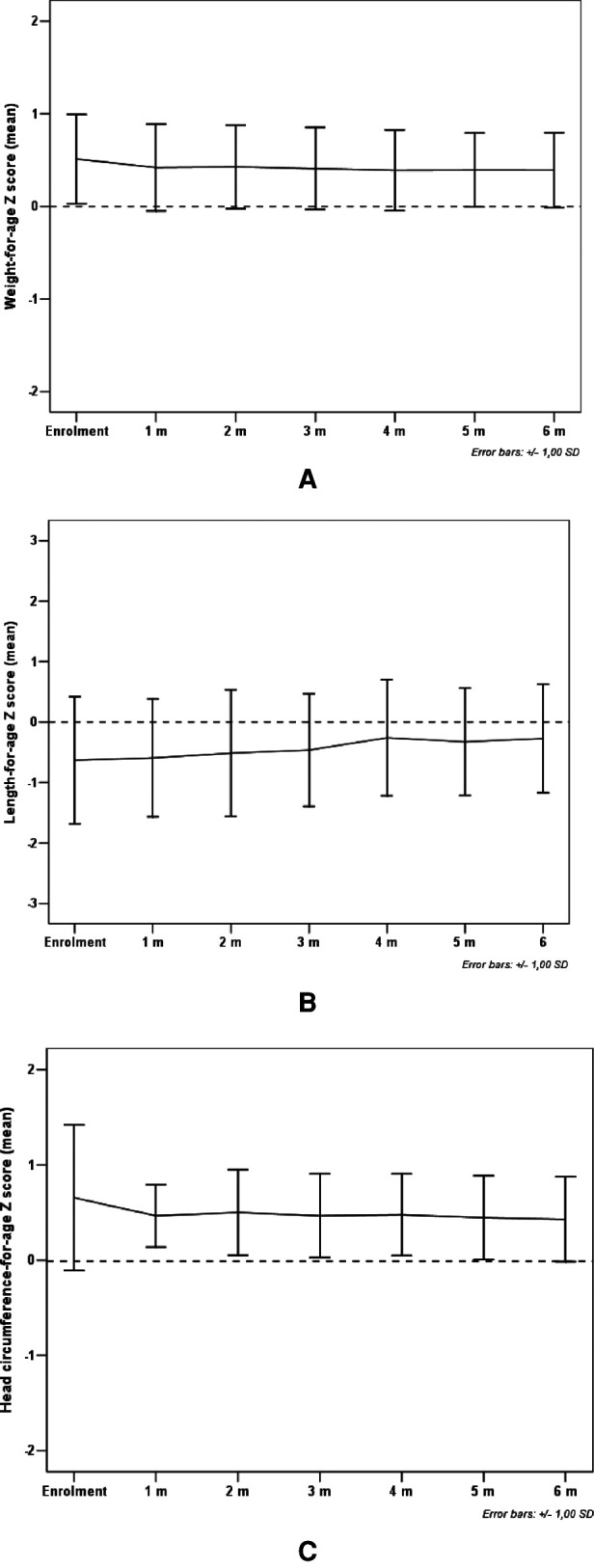
Mean weight-for-age Z score (panel **A**), length-for-age Z score (panel **B**) and head circumference-for-age Z score (panel **C**) from enrolment during 6-m period follow-up. Error bars indicate ±1 standard deviation.

All study subjects [80% male; 53.3% born with spontaneous delivery; 80% with familial allergy risk; mean age (±SD) at CMPA diagnosis 3 ± 2.5 months; mean age (±SD) at enrolment 16.7 ± 5.9 months, mean total serum IgE (±SD) 298.2 ± 200.4 kU/L] were from families of middle socioeconomic status and lived in urban areas. At baseline, all subjects were weaned and were receiving the new AAF. All 15 patients completed the 6-month study period and anthropometric measurements were available for all subjects. As depicted in the Table 2A, for the entire CMPA pediatric patients' cohort, from baseline to the end of the study period, a body growth pattern within normal range of WHO growth references was observed, as also suggested by the comparison with the healthy controls anthropometric values (Table 2B). The Figure 2 reported age-adjusted mean Z scores for body weight (panel A), length (panel B), and HC (panel C) calculated according to the WHO growth reference. The group means for WAZ, LAZ, and HCAZ tracked close to 0, confirmed that subjects maintained normal growth from enrolment to 6-m follow-up period.

**Table 2A:** Anthropometric measurements of subjects with cow’s milk allergy during the study.

Time point	*N*	Weight (kg)	WAZ	Length (cm)	LAZ	HC (cm)	HCAZ
Total cohort							
Enrolment	15	11.3 ± 1.7	0.51 ± 0.48	79.3 ± 5.7	−0.63 ± 1.05	47.7 ± 1	0.65 ± 0.76
1 m	15	11.5 ± 1.7	0.42 ± 0.47	80.4 ± 5.5	−0.59 ± 0.97	47.8 ± 1	0.47 ± 0.33
2 m	15	11.7 ± 1.6	0.43 ± 0.45	81.5 ± 5.5	−0.51 ± 1	48 ± 1.1	0.50 ± 0.45
3 m	15	11.9 ± 1.6	0.41 ± 0.44	82.7 ± 5.4	−0.46 ± 0.9	48.1 ± 1	0.46 ± 0.44
4 m	15	12.1 ± 1.6	0.39 ± 0.44	84.1 ± 5.3	−0.26 ± 1	48.3 ± 1	0.47 ± 0.43
5 m	15	12.3 ± 1.6	0.39 ± 0.4	85 ± 5.2	−0.33 ± 0.89	48.4 ± 0.9	0.44 ± 0.44
6 m	15	12.5 ± 1.6	0.39 ± 0.4	86 ± 5.2	−0.27 ± 0.9	48.5 ± 0.93	0.43 ± 0.45
Male subjects							
Enrolment	12	11.5 ± 1..6	0.49 ± 0.52	79.5 ± 5.2	−0.73 ± 1.1	47.6 ± 1.1	0.44 ± 0.51
1 m	12	11.6 ± 1.6	0.44 ± 0.5	80.6 ± 5.1	−0.68 ± 0.96	47.8 ± 1	0.38 ± 0.27
2 m	12	11.8 ± 1.6	0.42 ± 0.5	81.6 ± 5.1	−0.65 ± 0.98	48 ± 1	0.36 ± 0.26
3 m	12	12 ± 1.6	0.42 ± 0.48	82.7 ± 5	−0.57 ± 0.87	48.1 ± 0.9	0.34 ± 0.3
4 m	12	12.2 ± 1.6	0.40 ± 0.47	84.2 ± 4.9	−0.34 ± 0.94	48.3 ± 0.9	0.36 ± 0.28
5 m	12	12.4 ± 1.6	0.39 ± 0.44	85.1 ± 4.9	−0.44 ± 0.82	48.4 ± 0.9	0.31 ± 0.26
6 m	12	12.6 ± 1.5	0.38 ± 0.44	85.9 ± 4.9	−0.41 ± 0.78	48.5 ± 0.8	0.3 ± 0.27
Female subjects							
Enrolment	3	10.6 ± 2.1	0.59 ± 0.3	78.4 ± 8.5	−0.24 ± 0.88	47.9 ± 0.91	1.51 ± 1.13
1 m	3	10.8 ± 2	0.33 ± 0.35	79.9 ± 8.3	−0.22 ± 1.15	47.5 ± 1.2	0.81 ± 0.34
2 m	3	11.9 ± 2	0.42 ± 0.25	81.2 ± 8.1	0.02 ± 1.4	47.9 ± 1.8	1.05 ± 0.67
3 m	3	11.3 ± 2	0.36 ± 0.31	82.4 ± 8.1	−0.05 ± 1.2	48.1 ± 1.6	0.98 ± 0.63
4 m	3	11.5 ± 1.97	0.35 ± 0.32	83.7 ± 7.8	0.07 ± 1.2	48.2 ± 1.6	0.92 ± 0.68
5 m	3	11.8 ± 1.8	0.41 ± 0.23	84.9 ± 7.8	0.14 ± 1.19	48.4 ± 1.55	0.97 ± 0.69
6 m	3	12 ± 1.9	0.42 ± 0.21	86.2 ± 7.7	0.29 ± 1.3	48.6 ± 1.5	0.96 ± 0.69

Values are presented as mean ± standard deviation. WAZ, weight-for-age z-score; LAZ, length-for-age z-score; HCAZ, head-circumference-for-age z-score.

**Table 2B:** Anthropometric measurements of healthy controls.

Time point	N	Weight (kg)	WAZ	Length (cm)	LAZ	HC (cm)	HCAZ
Total cohort							
Enrolment	15	11.3 ± 1.7	0.67 ± 0.43	79.8 ± 6.5	0.05 ± 0.77	47.2 ± 1.38	0.36 ± 0.45
1 m	15	11.4 ± 1.6	0.59 ± 0.43	80.4 ± 6.3	−0.10 ± 0.78	47.4 ± 1.34	0.35 ± 0.42
2 m	15	11.6 ± 1.6	0.55 ± 0.42	80.9 ± 6.1	−0.23 ± 0.75	47.5 ± 1.33	0.32 ± 0.38
3 m	15	11.8 ± 1.5	0.54 ± 0.38	81.4 ± 6	−0.38 ± 0.69	47.7 ± 1.29	0.3 ± 0.38
4 m	15	12 ± 1.5	0.48 ± 0.37	81.9 ± 5.8	−0.55 ± 0.73	47.8 ± 1.27	0.28 ± 0.39
5 m	15	12.1 ± 1.4	0.45 ± 0.35	82.5 ± 5.8	−0.63 ± 0.79	48 ± 1.25	0.27 ± 0.38
6 m	15	12.3 ± 1.3	0.46 ± 0.36	83.1 ± 5.6	−0.71 ± 0.74	48.1 ± 1.26	0.28 ± 0.39
Male subjects							
Enrolment	12	11.6 ± 1.6	0.75 ± 0.44	80.4 ± 6.3	0.02 ± 0.84	47.5 ± 1.19	0.39 ± 0.49
1 m	12	11.7 ± 1.5	0.67 ± 0.44	80.9 ± 6.1	0.16 ± 0.86	47.7 ± 1.17	0.38 ± 0.45
2 m	12	11.9 ± 1.5	0.62 ± 0.44	81.4 ± 5.9	−0.3 ± 0.83	47.9 ± 1.16	0.36 ± 0.41
3 m	12	12.1 ± 1.5	0.6 ± 0.39	81.8 ± 5.7	−0.46 ± 0.76	48 ± 1.15	0.33 ± 0.41
4 m	12	12.2 ± 1.4	0.54 ± 0.39	82.2 ± 5.6	−0.65 ± 0.78	48.1 ± 1.14	0.31 ± 0.42
5 m	12	12.4 ± 1.4	0.5 ± 0.37	82.7 ± 5.8	−0.74 ± 0.83	48.3 ± 1.11	0.3 ± 0.42
6 m	12	12.5 ± 1.4	0.46 ± 0.35	83.3 ± 5.4	−0.84 ± 0.73	48.4 ± 1.11	0.32 ± 0.43
Female subjects							
Enrolment	3	10.1 ± 1.8	0.37 ± 0.14	77.6 ± 8.1	0.19 ± 0.47	45.8 ± 1.33	0.24 ± 0.28
1 m	3	10.2 ± 1.8	0.28 ± 0.1	78.4 ± 7.9	0.17 ± 0.17	46 ± 1.24	0.2 ± 0.29
2 m	3	10.4 ± 1.6	0.27 ± 0.12	79.2 ± 7.8	0.06 ± 0.05	46.1 ± 1.18	0.16 ± 0.18
3 m	3	10.6 ± 1.5	0.29 ± 0.12	79.8 ± 7.9	−0.08 ± 0.18	46.4 ± 1.07	0.17 ± 0.25
4 m	3	10.8 ± 1.4	0.26 ± 0.15	80.7 ± 8	−0.16 ± 0.32	46.5 ± 1.01	0.15 ± 0.18
5 m	3	11 ± 1.3	0.25 ± 0.19	81.5 ± 7.9	−0.21 ± 0.45	46.7 ± 1.06	0.14 ± 0.07
6 m	3	11.5 ± 1.1	0.44 ± 0.45	82.4 ± 7.8	−0.21 ± 0.59	46.8 ± 1.04	0.12 ± 0.07

Values are presented as mean ± standard deviation. WAZ, weight-for-age z-score; LAZ, length-for-age z-score; HCAZ, head-circumference-for-age z-score.

For all study subjects, sex- and age-related energy intake was checked at each study visit by experienced dieticians. For all study subjects total daily energy intake was within the recommended energy requirements for sex and age (24). Adherence to study formula was judged optimal for all study subjects. The daily mean (±SD) volume AAF intake was 281.3 ± 49.3 ml. Regarding safety data, there were 3 non-serious AEs due to respiratory infection (*n* = 1), febrile illness/viral infection (*n* = 1), acute gastroenteritis (*n* = 1). All AEs were deemed to be unrelated to the study formula.

## Discussion

This is the first study investigating the effects of this new AAF on body growth pattern of pediatric patients with IgE-mediated CMPA. We found that this new AAF promotes a normal body growth pattern.

The results of this study are well in line with other evidence demonstrating that infants with CMPA fed with an AAF presented adequate growth (25–29). In contrast, a recent review of growth patterns in healthy infants aged less than 4 months raised concerns that AAF treatment could be associated with suboptimal growth (30). However, our study design did not allow for a detailed assessment of the growth parameters in infants aged less than 4 months.

This study presents several strengths. First, it was performed on a well-characterized population of children with previous challenge-proven IgE-mediated CMPA followed by specialists at a tertiary paediatric allergy centre. Second, the methodology adopted in this study was rigorous, and diet and formula intake were assessed systematically.

Nonetheless, this study has limitations. Our data cannot be generalized to children with conditions that were reasons for exclusion from the study. Another limitation of our study is the lack of a control group fed with other hypoallergenic formula or a different AAF. These data could be potentially important because previous studies have suggested that CMPA children treated with AAF presented a suboptimal energy intake and a faltering growth (31, 32). Another limitation of our study is the lack of results of the longer evaluation of body growth pattern. But it should be underlined that the composition of the new AAF was fully in line with the composition of other commercially available AAFs and with the actual recommendation for energy requirement provided by the European Food Safety Authority (33). Thus, we can assume that this new AAF could support normal body growth in CMPA children also in the long term. However, to better assess this aspect, future studies to assess the long-term effects of AAF on growth and body composition are advocated.

In conclusion, the new AAF ensured normal growth in pediatric patients affected by IgE-mediated CMPA and it could be a suitable safe option, among the special formulas already available, for the dietary management of children affected by this form of food allergy.

## Data Availability

The raw data supporting the conclusions of this article will be made available by the corresponding author on reasonable request.
